# PSA-selective activation of cytotoxic human serine proteases within the tumor microenvironment as a therapeutic strategy to target prostate cancer

**DOI:** 10.18632/oncotarget.25091

**Published:** 2018-04-27

**Authors:** Oliver C. Rogers, Lizamma Anthony, D. Marc Rosen, W. Nathaniel Brennen, Samuel R. Denmeade

**Affiliations:** ^1^ Department of Pharmacology and Molecular Sciences, Johns Hopkins Medical Institutions, Baltimore, MD, USA; ^2^ Department of Oncology, Johns Hopkins Medical Institutions, Baltimore, MD, USA

**Keywords:** granzyme, trypsin, PSA, prostate, metastatic

## Abstract

Prostate cancer is the most diagnosed malignancy and the second leading cause of cancer-related death in American men. While localized therapy is highly curative, treatments for metastatic prostate cancer are largely palliative. Thus, new innovative therapies are needed to target metastatic tumors. Prostate-Specific Antigen (PSA) is a chymotrypsin-like protease with a unique substrate specificity that is secreted by both normal and malignant prostate epithelial cells. Previous studies demonstrated the presence of high levels (μM-mM) of enzymatically active PSA is present in the extracellular fluid of the prostate cancer microenvironment. Because of this, PSA is an attractive target for a protease activated pro-toxin therapeutic strategy. Because prostate cancers typically grow very slowly, a strategy employing a proliferation-independent cytotoxic payload is preferred. Recently, it was shown that the human protease Granzyme B (GZMB), at low micromolar concentrations in the extracellular space, can cleave an array of extracellular matrix (ECM) proteins thus perturbing cell growth, signaling, motility, and integrity. It is also well established that other human proteases such as trypsin can induce similar effects. Because both enzymes require N-terminal proteolytic activation, we propose to convert these proteins into PSA-activated cytotoxins. In this study, we examine the enzymatic and cell targeting parameters of these PSA-activated cytotoxic serine proteases. These pro-enzymes were activated robustly by PSA and induced ECM damage that led to the death of prostate cancer cells *in vitro* thus supporting the potential use of this strategy as means to target metastatic prostate cancers.

## INTRODUCTION

More than 165,000 men will be diagnosed annually in the US with prostate cancer [1]. Despite notable success in managing patients with localized disease, ~29,000 men will ultimately succumb to prostate cancer this year [1]. While considerable progress has been made, current therapies for metastatic prostate cancer (MPC) produce only a modest survival benefit and provide only temporary relief from disease burden. Thus, it is vital that new therapeutic strategies are developed to combat this malignancy.

An important characteristic of prostate cancer that must be taken into consideration when developing new therapeutics is its remarkably low proliferative rate that makes prostate cancer among the slowest growing of the solid tumors [2]. This low proliferative rate is seen in both newly diagnosed and castrate resistant prostate cancers. Because the vast majority of prostate cancer cells within a given site are proliferatively quiescent in the G0 phase of the cell cycle [2], a preferred therapeutic strategy would be to develop new agents that kill prostate cancer cells in a proliferation independent manner.

A further characteristic of all stages of prostate cancer, including high grade castration resistant cancer, is the secretion of high levels of the enzymatically active serine protease Prostate-specific Antigen (PSA) in the extracellular fluid (ECF) within the tumor microenvironment [3, 4]. PSA is aptly named as it is only expressed to any significant degree by normal and malignant prostate epithelial cells within the human [5, 6]. While PSA is highly active in the seminal fluid and in the ECF of prostate cancers [3–6], it is inactivated in the bloodstream of patients due to the covalent binding to the abundant serum protease inhibitors α-1-antichymotrypsin and α-2-macroglobulin [7, 8]. Therefore active PSA is only present in the prostatic fluid and in the ECF of sites of prostate cancer. Thus, PSA is an attractive target for a protoxin based therapeutic strategy in which an inactive proliferation-independent cytotoxin would only be activated by PSA selectively within the tumor microenvironment [9]. An additional benefit to this strategy is the potential bystander effect by which non-PSA producing stromal and endothelial cells in the tumor microenvironment could also be killed following drug activation within the ECF. Previously we validated this PSA-activated protoxin approach using the bacterial protoxin proaerolysin [9]. This agent is currently in Phase I-III testing as intraprostatic therapy for localized prostate cancer and benign prostatic hyperplasia [10, 11].

While many immunotoxin and protoxin approaches have been described, these strategies typically involve the use of non-human protein toxins. The immunogenicity of these toxins has limited their use as systemic antitumor agents due to rapid development of neutralizing antibodies that limit or prevent repeat dosing. This obstacle could potentially be overcome through the use of human protoxins that could be toxic if activated in an inappropriate cellular compartment such as the ECF of prostate cancer sites. In this study, we describe a strategy to reengineer the pro-apoptotic proteases Granzyme B (GZMB) and Trypsin for activation by PSA within the prostate cancer ECF. Granzyme B is a secreted serine protease expressed by activated Cytotoxic T-Lymphocytes (CTLs) [12]. When co-secreted into the immune synapse along with the pore-forming protein perforin, GZMB can translocate into the target cell cytoplasm, cleave and activate pro-apoptotic protein, and induce target cell death. Intracellular-substrates of GZMB include members of the caspase family, ICAD, BCL-2 family proteins, and several pro-mitotic factors [13]. In addition to these intracellular effects, GZMB was shown to also be able to degrade various components of the extracellular matrix (ECM) at high nanomolar concentrations. This extra-cellular mechanism of GZMB was shown to induce anoikis of surrounding cells, decrease cell motility, and potentially act as a pro-inflammatory cytokine [14–17]. Unlike other serine proteases, extracellular GZMB is relatively resistant to inhibition by serum and tissue protease inhibitors suggesting it may have long-acting effects *in vivo*. Upon trafficking to the secretory granule of the CTL, pro-GZMB is activated by removal of an N-terminal dipeptide by Cathepsin C [18, 19]. Trypsin (TRP), has also been well characterized for its ability to cleave ECM components. Prolonged incubation of cancer cells with TRP is well-known to induce cell death. Pro-TRP is the inactive zymogen that is activated by removal of the propeptide, DDDDK by enterokinase (EK) [20, 21]. Because both GZMB and TRP have extracellular effects on human cells *in vitro* and have well characterized pro-forms activated by serine proteases, they are interesting candidates as potential prostate-targeting PSA-activated pro-drugs.

**Figure 1:**
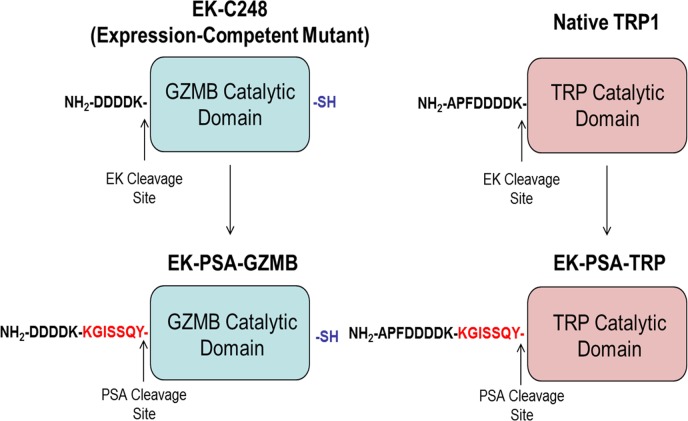
Schematic of EK-PSA-GZMB and EK-PSA-TRP pro-drugs Mutations made to convert EK-activated GZMB into a PSA-activated zymogen (left) and WT Trypsin into a PSA-activated zymogen (right).

Based on prior studies done with recombinant Granzyme B [22], we hypothesized that the replacement of the N-terminal pro-peptides of GZMB and TRP with a peptide substrate that is selectively and efficiency cleaved by PSA [4] would yield zymogens that would be inactive in normal tissues but efficiently activated by high levels of enzymatically active PSA present in the ECF of prostate cancer sites (Figure 1). Inappropriate activation of these proteases in the ECF will induce death of PSA-producing prostate cancer cells as well as non-PSA producing bystanders such as stromal and endothelial cells. In this study we describe the production of these human cytotoxic serine proteases as substrates for PSA and evaluate their ability to selectively kill PSA-producing cells *in vitro*.

## RESULTS

**Figure 2:**
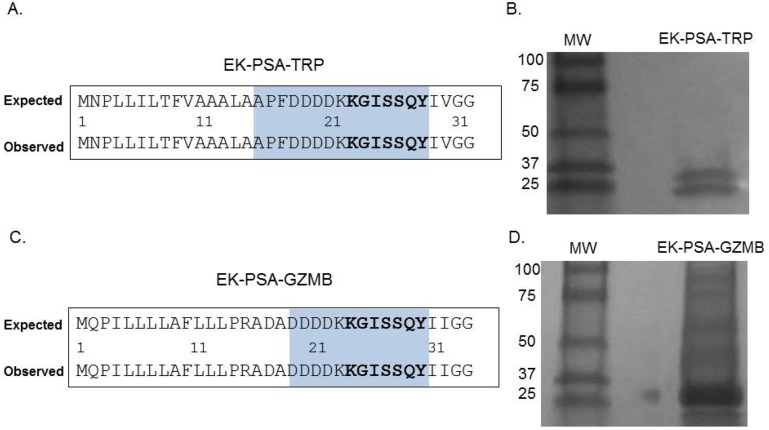
Confirmation of EK-PSA-TRP and EK-PSA-GZMB Results of Sanger sequencing **(A, C)** and Western blot analysis **(B, D)** for EK-PSA-TRP and EK-PSA-GZMB respectively.

To verify that all pro-enzymes constructs were produced correctly, Sanger sequencing was performed on both the PSA-activated TRP and GZMB genes. DNA sequencing analysis confirmed that both proteins contained an inserted KGISSQY PSA cut site immediately flanked by their respective catalytic domains and the inhibitory EK peptide (Figure 2A & 2C). For both mutants lacking their respective EK peptides, Sanger sequencing showed the successful removal of these residues thus yielding N-termini beginning with the KGISSQY substrate (data not shown). SDS PAGE analysis showed that both protein constructs were relatively pure following extraction from cell supernatant and further purification. EK-PSA-TRP protein when run under reducing conditions yielded two bands at 25 and 28 kDa (Figure 2B) while the GZMB mutant ran as a single band at 28 kDa (Figure 2D). Under non-reducing conditions EK-PSA-GZMB will run as both a monomer and a di-sulfide linked dimer (data not shown). Expression yields for both of the PSA-activated protease mutants lacking the N-terminal EK sequence (referred to as PSA-TRP and PSA-GZMB) were substantially lower. Yields did also not improve when using NiNTA chromatography on PSA-GZMB despite it being cloned with an N-terminal His tag.

**Figure 3:**
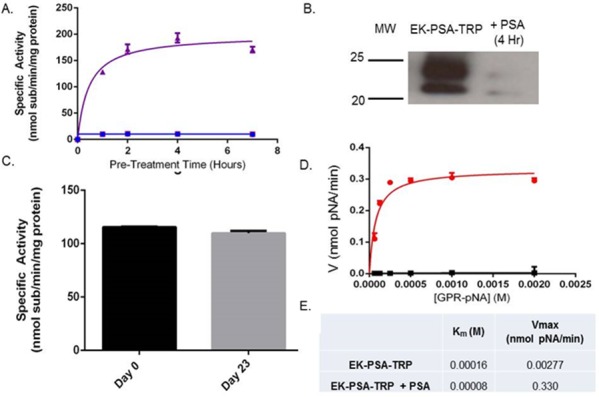
EK-PSA-TRP is a PSA-activated Trypsin mutant Enzymatic hydrolysis of GPR-pNA by EK-PSA-TRP (red), PSA, (blue), and EK-PSA-TRP mixed with PSA (purple) pre-incubated for various times **(A)**. Western blot analysis of EK-PSA-TRP +/- PSA probed with an anti-DDDDK rabbit polyclonal antibody **(B)**. Enzymatic activity of EK-PSA-TRP incubated for 0 or 23 days at 37 degrees after being pre-exposed to PSA **(C)**. Michaelis-Menten enzyme kinetics of EK-PSA-TRP (black) or EK-PSA-TRP with PSA (red) **(D)**. Calculated enzymatic parameters of inactive and activated EK-PSA-TRP **(E)**.

Following expression, harvesting, and NiNTA purification, we examined the enzymatic characteristics of the EK-PSA-TRP protein. Using a trypsin-specific chromogenic assay, we pre-incubated the drug plus or minus enzymatically active PSA. Purified PSA and EK-PSA-TRP alone were unable to cleave the GPR-pNA trypsin substrate. However, when pre-incubated together, we observed a steady increase in substrate hydrolysis that stabilized after roughly 4 hours (Figure 3A). Western blotting of these mixtures using an antibody specific for the EK sequence tag on the N-terminus showed only the uncleaved protein (running at both 25 and 28 kDa) with no PSA present. This signal disappeared after a 4 hour incubation with PSA (Figure 3B). Using the GPR-pNA release assay, we assessed the stability of the EK-PSA-TRP protein after a 4 hour pre-incubation with PSA. We observe no substantial loss of TRP enzymatic activity after a 23 day incubation at 37 degrees Celsius compared with Day 0 (Figure 3C). Velocity experiments confirmed that the active EK-PSA-TRP mutant displays classical Michaelis-Menten enzyme kinetics with linear changes in velocity below 500 μM substrate (Figure 3D). While some baseline activity of EK-PSA-TRP in the absence of PSA was noted, the Vmax between the PSA-treated and non-treated protein differed by almost 120-fold while the Km values were not notably different.

**Figure 4:**
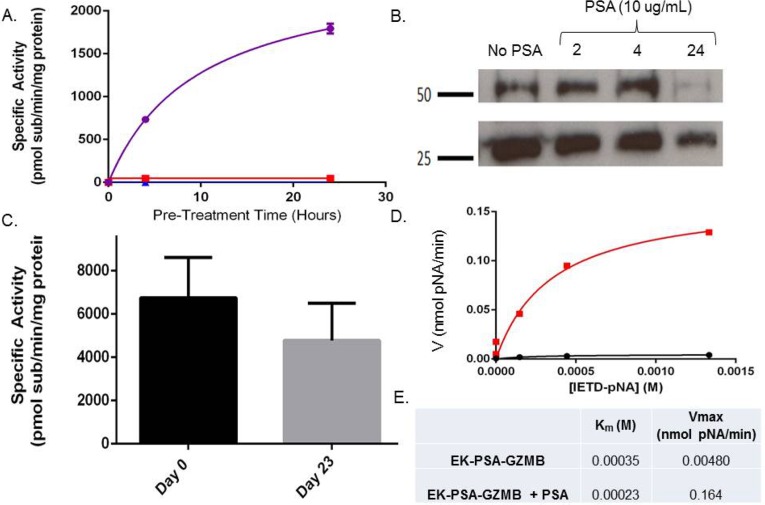
EK-GZMB-TRP is a PSA-activated Granzyme B mutant Enzymatic hydrolysis of GPR-pNA of EK-PSA-GZMB (red), PSA (blue), and EK-PSA-TRP mixed with PSA (purple) pre-incubated for various times **(A)**. Western blot analysis of EK-PSA-GZMB +/- PSA probed with an anti-DDDDK rabbit polyclonal antibody **(B)**. Enzymatic activity of EK-PSA-GZMB incubated for various time points at 37 degrees after being pre-exposed to PSA **(C)**. Michaelis-Menten enzyme kinetics of EK-PSA-GZMB (black) or EK-PSA-TRP with PSA (red) **(D)**. Calculated enzymatic parameters of inactive and activated EK-PSA-GZMB **(E)**.

In subsequent studies, we evaluated the enzymatic activity of EK-PSA-GZMB in terms of activation kinetics, stability, and catalytic kinetics. Using a fluorescent substrate for GZMB, IETD-AFC, we assessed the activity of EK-PSA-GZMB with or without PSA pre-treatment following various pre-incubation times. Purified PSA and the EK-PSA-GZMB had very low to no activity against this substrate. (Figure 4A). However, PSA-treated EK-PSA-GZMB showed robust enzymatic processing of the GZMB substrate which reached a plateau at 20 to 24 hours. Western Blot of the PSA treated EK-PSA-GZMB reactions using non-reducing conditions showed a decrease on both monomer and dimer bands of the protein with a vast majority of the signal disappearing after 24 hours of incubation with PSA (Figure 4B). Stability studies of activated EK-PSA-GZMB showed that, similar to the EK-PSA-TRP protein, the GZMB enzyme retained most of its enzymatic activity after 23 days at 37 degrees (Figure 4C). Enzyme kinetic studies using the IETD-pNA substrate demonstrated a 30-fold difference in Vmax between the “inactive” vs. PSA treated EK-PSA-GZMB with relatively no difference in Km. Similar to the EK-PSA-TRP, the enzyme's linear change in velocity was observed at concentrations less than 0.5mM substrate. (Figure 4E). To confirm PSA cleavage was occurring at the desired locus on the protein, we isolated PSA-digested EK-PSA-GZMB and performed Edman degradation. N-terminal analysis of the protein confirmed that the residues I-I-G were the first three amino acids of the protein (data not shown) consistent with that of the active GZMB enzyme.

**Figure 5:**
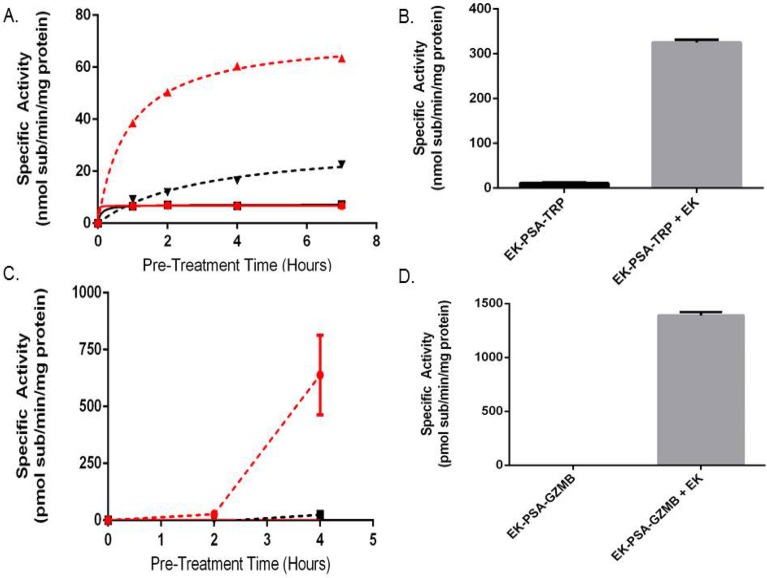
The N-terminal EK piece both stabilizes and inhibits EK-PSA-TRP and EK-PSA-GZMB Enzymatic activity of pre-incubated EK-PSA-TRP + PSA (red dashed), PSA-TRP + PSA (black dashed), EK-PSA-TRP alone (red solid), and PSA-TRP alone (black solid) **(A)**. Enzymatic activity of EK-PSA-TRP +/- a 4 hour EK treatment **(B)**. Enzymatic activity of pre-incubated EK-PSA-GZMB + PSA (red dashed), PSA-GZMB + PSA (black dashed), EK-PSA-GZMB alone (red solid), and PSA-GZMB alone (black solid) **(C)**. Enzymatic activity of EK-PSA-GZMB +/- a 4 hour EK treatment **(D)**.

In order to more completely understand the mechanism behind the mutations made to modify the zymogen activation of TRP and GZMB, we removed the EK sequence from each protein (native TRP and EK-GZMB) and inserted the PSA-activated substrate KGISSQY yielding mutants PSA-TRP and PSA-GZMB. Activation kinetic assays with PSA-TRP and EK-PSA-TRP showed activation of both proteins following a four hour PSA incubation. However, PSA-activated EK-PSA-TRP demonstrated 4 fold more enzymatic activity compared to PSA-TRP (Figure 5A). Similar results were observed with the GZMB constructs with EK-PSA-GZMB showing substantial more activity than PSA-GZMB following 4 hour incubation with PSA (Figure 5A & 5C). In the case of GZMB, the PSA-GZMB species had no detectable activity compared to the somewhat active PSA-TRP. Both EK-PSA-TRP and EK-PSA-GZMB proteins, when treated with porcine EK at 5 EU/mL were both robustly active after a four hour incubation (Figure 5B & 5D). Both active enzymes had a higher specific activity following incubation with EK than with PSA.

**Figure 6:**
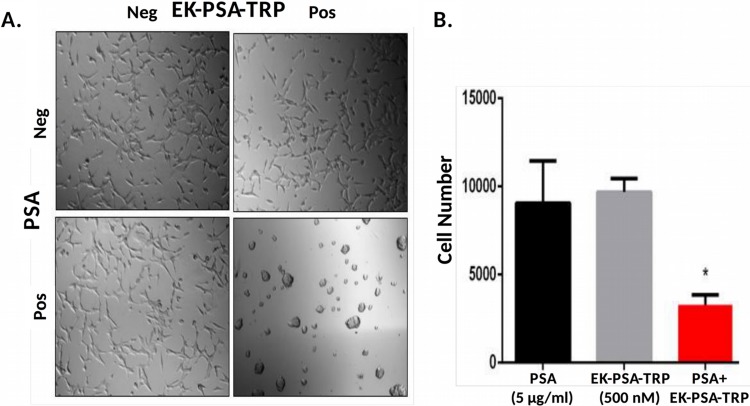
EK-PSA-TRP inhibits LNCaP cell growth selectively in the presence of PSA Light microscopy images of LNCaP cells treated +/- 500 nM EK-PSA-TRP and +/- PSA at 10X magnification **(A)**. MTT assay of LNCaP cells after 5 days treatment with PSA-containing media (5 μg/ml) (black bar) or 500 nM EK-PSA-TRP in media (grey bar) or both (red bar) **(B)**.

In order to accurately mimic the prostate cancer microenvironment and evaluate any effect these proteins have on cell growth, we plated various cell types on plastic tissue culture plates pre-treated with media containing 10% serum. The cells were then allowed to attach to the dish and remain viable in media lacking serum protease inhibitors that would otherwise disrupt PSA's ability to activate these drugs. EK-PSA-TRP at 500 nM was treated on LNCaP cells plus or minus exogenously added PSA. Cells treated with the vehicle alone grew slower than under normal conditions but were otherwise healthy and viable (Figure 6A). LNCaP cells exposed to either PSA alone or EK-PSA-TRP alone were unaffected. However, when both were co-incubated on cells, we observe gross morphology and violent aggregation consistent with how LNCaP cells grow with no ECM present (Figure 6A). Using the MTT assay and an LNCaP standard curve, we were able to quantitate the cell number for each treated group. Over the period of 5 days, we observe that both groups treated with EK-PSA-TRP or PSA alone doubled in cell count which was not substantially different from the control cells. LNCaP cells affected by activated TRP digestion of the ECM did not grow at all, as number of cells plated and counted cells were identical. This difference in cell number was found to be statistically significant (p <.05). This experiment was repeated on DU145, CWR22 Rv1, and prostate stromal cells donated from healthy patients and we observed no notable differences in morphology or cell growth between treated and untreated cells (data not shown).

**Figure 7:**
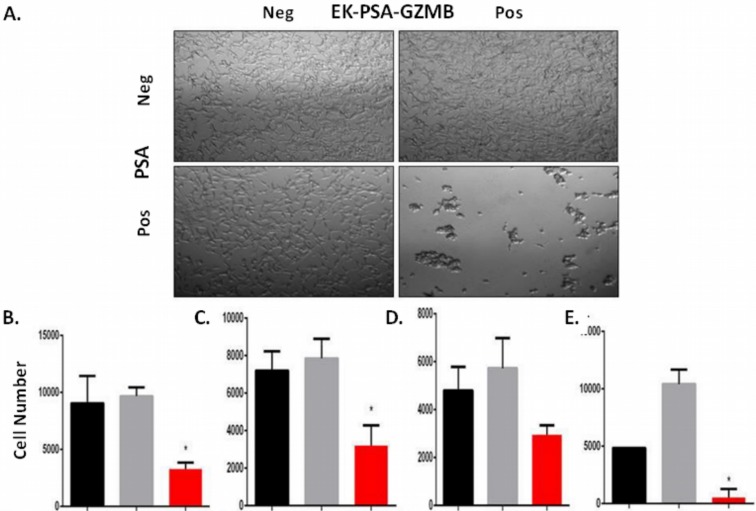
EK-PSA-TRP inhibits prostate cell growth selectively in the presence of PSA Light microscopy images of LNCaP cells treated +/- 3 uM EK-PSA-GZMBand +/- PSA at 10X magnification **(A)**. MTT assay after 5 days of treatment of LNCaP **(B)**, DU145 **(C)**, CWR22 Rv1 **(D)**, normal human prostate stromal cells **(E)** with PSA-containing media (5 μg/ml) (black bar) or 500 nM EK-PSA-TRP in media (grey bar) or both (red bar).

We performed the same set of experiments to assess EK-PSA-GZMB's ability to inhibit the growth of prostate cancer cells and if the toxicity was limited to just LNCaP cells. For the light microscopy experiment, we observed, again, no effect of the cells treated with EK-PSA-GZMB or PSA alone. In combination, 3 μM EK-PSA-GZMB activated by PSA induced obvious morphology changes and clumping of affected cells (Figure 7A). To further understand these effects kinetically, we used time lapse microscopy and observed that a majority of the clumping occurs within 24 hours of treatment with the activated GZMB protein (data not shown). Like the EK-PSA-TRP drug, these effects correlated with a significantly significant difference in growth, with cells treated with PSA and EK-PSA-GZMB not appearing to grow when compared to controls and the starting number of cells plated on Day 0 (Figure 7B). Unlike the EK-PSA-TRP protein, however, we did observe both a morphological and anti-proliferative effect on DU145, CWR22 Rv1, and stromal cells. When treated with PSA or EK-PSA-GZMB alone, no morphological differences were observed on any of these cells. PSA-treated EK-PSA-GZMB induced cell aggregation, morphological changes and differences in cell adhesion to the plate (data not shown). Like the LNCaP treated with EK-PSA-GZMB, we observe a growth inhibitory effect on each cell line when compared with the respective controls on DU145, and CWR22 Rv1 cells (Figure 7C and 7D). To demonstrate the potential for a bystander effect we also demonstrated that EK-PSA-GZMB was non-toxic against normal prostate stromal cells in the absence of PSA but these cells were readily killed by EK-PSA-GZMB in the presence of PSA, (Figure 7E). These results were found to be statistically significant (p<0.05). Interestingly, GZMB at this dose induced morphological effects on PC3 and LAPC4 cells but no changes in cell count was seen (data not shown).

**Figure 8:**
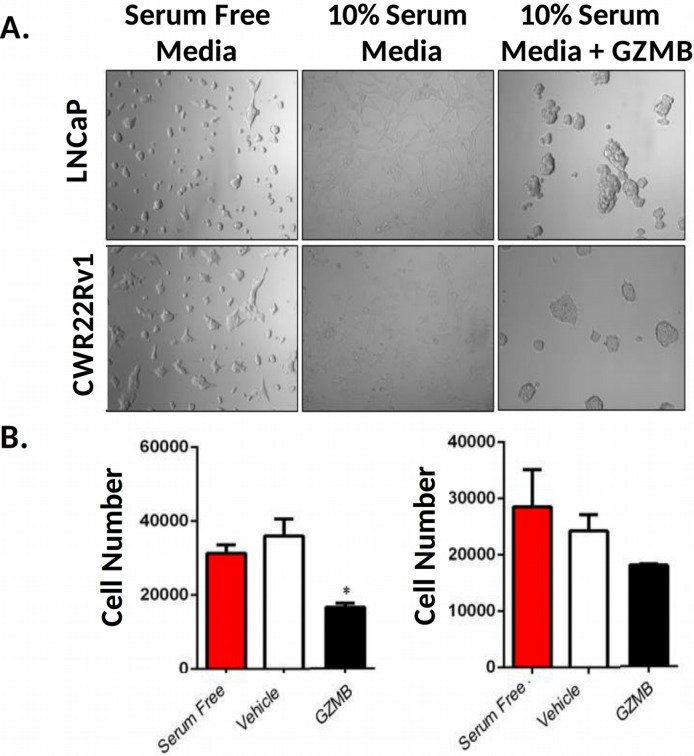
GZMB induces prostate cancer cell growth inhibition via damage to the ECM Light microscopy images of LNCaP (top) and CWR22 Rv1 (bottom) were plated in serum free RPMI (B27 supplemented), in wells treated with buffer (left), media-containing serum (middle) or media with serum and 1 uM GZMB (right) and incubated for 48 hours at 37 degrees. Magnification is 10X **(A)**. Quantitation of plated LNCaP (left) and CWR22 Rv1 (right) 48 hours after plating **(B)**.

Because we observed notable effects on cell morphology and growth which strongly correlated with enzymatic activity of these drugs, we aimed to examine whether these effects were driven by protease-induced ECM damage. To do this, we pre-coated a plate with serum free media, media containing 10% serum, or media with serum and 1 μM GZMB. We then removed the solutions and plated LNCaP and CWR22 Rv1 cells in serum free media and incubated them for 48 hours. Cells plated on surfaces coated with media attached well, had normal morphology, and proliferated (Figure 8A). Cells plated in serum free wells did not attach but appeared to grow at rates comparable to the control. Both LNCaP and CWR22 Rv1 cells plated on wells treated with serum and GZMB displayed morphology similar to those treated with activated EK-PSA-GZMB and did not grow as well when quantitated with the MTT assay (Figure 8B). Only the growth of the LNCaP cells were significantly affected by the GZMB treated matrix while the CWR22 Rv1 cells were not despite the obvious visual effects.

## DISCUSSION

The primary goal of this study was to determine if human proteases which are not toxic in their normal physiologic context could be reengineered to produce cytotoxicity when activated in the tumor microenvironment. We selected Granzyme B because it is not cytotoxic in the T-cell granule but can induce apoptosis following intracellular uptake by targeted cells. Similarly, trypsin is not toxic following physiologic activation within the small intestine. However, it is well recognized that failure to neutralize trypsin in tissue culture experiments results in death of overexposed cells within a short time period. Both of these enzymes are capable of degrading components of the extracellular matrix (ECM). Previous studies have demonstrated that ECM degradation can activate apoptosis *in vitro* and *in vivo*. Human cancer cells interact with the ECM and thus, targeted disruption of the ECM can inhibit cell growth and lead to cell death. This is an attractive mode of therapeutic targeting as it can overcome tumor cell heterogeneity because it affects the tumor microenvironment shared by all tumor cells rather than just targeting cells expressing a specific marker.

Previously we have demonstrated that a potent bacterial toxin proaerolysin could be reengineered to be activated in the prostate cancer microenvironment through the proteolytic activity of the serine protease PSA [9]. We further demonstrated that PSA is present in high concentrations in the ECF in an enzymatically active form but becomes inactivated upon entering the circulation due to binding to serum protease inhibitors [3, 4]. PSA-activated proaerolysin is currently in clinical development as an intraprostatic therapy for BPH and prostate cancer [10, 11]. However, proaerolysin is a bacterial toxin that is highly immunogenic producing neutralizing antibodies that would limit repeat dosing. Thus, a major advantage of retargeting human proteins compared to all other approaches using non-human bacterial or animal derived protein toxins is that this immunogenicity problem can be overcome.

We demonstrated that PSA activated forms of both human TRP and GZMB could be generated by replacing the wild type propeptide piece of the zymogen with a 6 amino acid sequence that is a specific substrate recognized by PSA. Using a TRP or GZMB specific substrate we show that both mutants, EK-PSA-TRP and EK-PSA-GZMB, remain enzymatically inactive until incubated with PSA. Interestingly, we observe very different activation kinetics of both proteases based on the plateau of the enzymatic assay and the immunoblots probing with an anti-DDDDK antibody. The EK-PSA-TRP zymogen was activated at least 5 times faster than the EK-PSA-GZMB, suggesting that the PSA propeptide may be presented in a more favorable conformation on the TRP protein possibly due to the presence of the wild type EK propeptide in the sequence. Both EK-PSA propeptide containing protease showed less enzymatic activity per mg enzyme than their EK-propeptide only counterparts that were activated by enterokinase alone. These results suggest that the presence of the PSA propeptide may adversely affect protein folding. However, both EK-PSA-TRP and EK-PSA-GZMB retained a majority of their initial activity following a 23 day incubation at 37 degrees Celsius suggesting their stability would not be a limitation for use as an *in vivo* therapy.

To further explore the role of both peptides (DDDDK and KGISSQY) within the prodomain we generated two more mutants containing only the KGISSQY PSA substrate as the propeptide on the N-termini of TRP and GZMB deemed PSA-TRP and PSA-GZMB respectively. We then expressed these mutants in mammalian cells using the same methodology and found that both PSA-GZMB and PSA-TRP expressed at much lower levels than the EK-containing PSA-activated variants suggesting that the EK piece promotes proper folding and expression of these enzymes. Furthermore, PSA-TRP and PSA-GZMB had substantially less enzymatic activity when incubated with PSA for the same length of time as EK-PSA-TRP and EK-PSA-GZMB with PSA-GZMB having no detectable GZMB activity after a 4 hour incubation with PSA. This furthers indicates that the EK propeptide may play a role in the proper folding of the inactive zymogen to allow for fully functional enzyme once the EK peptide is proteolytically removed. The finding that the DDDDK propeptide appeared to promote proper folding, proper presentation of the KGISSQY peptide activation substrate, and direct inhibition of the enzymes when attached, consideration should be given to its inclusion in other applications that require selective activation of a target protease.

To fully characterize both EK-PSA-GZMB and EK-PSA-TRP's ability to target cells in culture both proteins were incubated with cells in serum free media to prevent PSA inactivation by serum protease inhibitors. However, cells were first plated in 10% FBS containing media to allow for attachment prior to transfer to serum free media. EK-PSA-GZMB produced obvious morphological effects and growth inhibition on all cell lines only when co-incubated with PSA. In contrast, EK-PSA-TRP only had effects on LNCaP cells in the presence of PSA but had no effect on the other cell lines tested. This is likely due to the fact that many cancer lines secrete anti-trypsin protease inhibitors as a mechanism to prevent extracellular damage via trypsin and trypsin like proteases [20, 21]. GZMB, on the other hand, has been shown to be resistant to inhibition via serum protease inhibitors which would explain the discrepancy in effect between these two proteins. EK-PSA-GZMB also inhibited growth of stromal fibroblast cells suggesting that following PSA activation in the tumor microenvironment, this protein has the potential to have a broad bystander effect on multiple cell types

To determine if GZMB's effects were due to ECM disruption, we precoated tissue culture plates coated with ECM proteins from FBS then exposed the plate to GZMB in a cell free context. Following GZMB removal, cells were plated in serum free media. We observed very similar morphologic and growth inhibitory effects on both LNCaP and CWR22Rv1 cell lines. Because the cell morphology was very similar to that of the cells co-incubated with PSA and EK-PSA-GZMB in co-culture experiments, we concluded that the effects seen were due to ECM disruption caused by GZMB proteolysis.

In conclusion, we successfully engineered two human proteases to be selectively activated by a cancer-specific protease PSA. These results demonstrate that these human proteases could be reengineered to create potentially therapeutic proteins that can selectively modify the tumor microenvironment to generate an anti-tumor response. These new human protein toxins have an advantage over non-human protein toxins as they are not immunogenic. Further studies are underway to optimize these human protease therapeutics for *in vivo* testing against prostate cancer xenografts.

Human cancer cells interact with the ECM and thus, targeted disruption of the ECM can inhibit cell growth and lead to cell death. This is an attractive mode of therapeutic targeting as it can overcome tumor cell heterogeneity because it affects the tumor microenvironment shared by all tumor cells rather than just targeting cells expressing a specific marker. Previously, we have explored the potential of prostate cancer specific proteases as targets to activate small molecule cytotoxins and protein protoxins. These studies build on that prior work and are aimed at exploring the idea of using proteases as both the targeting agent as well as the cytotoxic moiety. Dysregulated proteolytic activity in an inappropriate context can be toxic and is the basis of disease states such as pancreatitis and alpha-1-antitrypsin deficiency. In order to target the potential cytotoxicity of proteases to the tumor microenvironment and limit the effects on normal tissue, we sought to engineer these proteases into zymogen mutants capable of being activated selectively by PSA. To achieve this, we chose to modulate the zymogen activation of human TRP, as it has been well established to digest components of the ECM and has a well characterized zymogen activation. Our mutant, EK-PSA-TRP, contained a robustly cleaved PSA substrate, KGISSQY flanked by the inhibitory EK substrate and the rest of the enzyme. This protein was successfully expressed in mammalian tissues, albeit at rather low levels due to toxic effects observed on the HEK293T cells. We also chose human GZMB as a second candidate due to its published ability to digest the ECM, to be expressed as a zymogen with an EK piece on the N-terminus, and to resist inhibition via serum protease inhibitors. This protein was easily expressed in mammalian cells.

We have successfully engineered both human TRP and GZMB to be activated by PSA. Incubation experiments with PSA using a TRP and GZMB specific substrate has shown that both mutants, EK-PSA-TRP and EK-PSA-GZMB, remain inactive until incubated with PSA. Interestingly, we observe very different activation kinetics of both drugs based on the plateau of the enzymatic assay and the immunoblots probing with an anti-DDDDK antibody. Because the TRP zymogen was activated at least 5 times faster than the GZMB, it suggests the PSA substrate was presented in a more favorable conformation on the TRP pro-drug possibly due to the EK piece already being in a more native orientation. Both pro-drugs showed less activity per mg enzyme than their EK-activated counterparts suggesting that the PSA substrate peptide affects the folding (data not shown) of the enzymes negatively. We were, however, pleased to see that both enzymes retained a majority of their initial activity following a 23 day incubation at 37 degrees Celsius suggesting their stability would not be a limitation for use as an *in vivo* therapy. We were also pleased to see the robust difference in Vmax values for both enzymes compared to their zymogen counterparts while seeing very little change in Km suggesting that PSA activation and removal of the EK inhibitory piece induced a conformational change in the zymogen that increased enzymatic turnover as opposed to changing substrate binding.

The simple insertion of the KGISSQY PSA substrate into the native TRP and EK-GZMB proteins to yield a PSA-activated mutant was unprecedentedly simple. We chose to further delve into the role of both peptides (DDDDK and KGISSQY) in yielding such a mutant given that EK-GZMB and EK-TRP have already been established as EK activated zymogens. To do this, we designed two more mutants containing only the KGISSQY substrate on the N-termini of TRP and GZMB deemed PSA-TRP and PSA-GZMB respectively. We then expressed these mutants in mammalian tissues and used the same chromatography methods as the EK-containing proteins. It should be noted that, in terms of expression, both PSA-GZMB and PSA-TRP expressed at much lower levels than the EK-containing PSA-activated variants suggesting that the EK piece promotes proper folding and expression of these enzymes. Furthermore, PSA-TRP and PSA-GZMB had substantially less activity when incubated with PSA at the same length as EK-PSA-TRP and EK-PSA-GZMB with PSA-GZMB having no detectable activity after a 4 hour incubation with PSA. This also indicates that the EK piece plays a role in the proper folding of the inactive zymogen so the functional enzyme can be released once the EK peptide is proteolytically removed. Because EK also robustly activated EK-PSA-TRP and EK-PSA-GZMB robustly, it also strongly suggests that the DDDDK peptide also plays a major role in inhibiting the enzymes and retaining the inactive conformation, possibly due to the very strong ionic charge and secondary structure formed by the piece. It is interesting that the PSA-TRP protein retained some activity once activated by PSA as opposed to the PSA-GZMB protein suggesting the TRP is more tolerant about which peptides reside on its N-terminus for proper expression and folding. Because the DDDDK EK piece seems to promote folding, proper presentation of the KGISSQY peptide activation substrate, and direct inhibition of the enzymes when attached, we highly recommend it's use as a pro-piece for any further mutant serine proteases that require activation by a target protease.

We fully characterize both EK-PSA-GZMB and EK-PSA-TRP's ability to target cells in culture. We assessed this by incubating both drugs in serum free media (to facilitate PSA activation) on cells plated on a bed of matrix proteins derived from FBS. On LNCaP prostate cancer cells, we observed obvious morphological and proliferative effects from both drugs only when PSA and the pro-drug were co-incubated. While EK-PSA-GZMB had these effects on various other prostate cancer cell lines to a varying degree, we did not observe these effects on any other cell line when using EK-PSA-TRP. This is likely due to the fact that many cancer lines secrete anti-trypsin protease inhibitors as a mechanism to prevent extracellular damage via trypsin and trypsin like proteases. GZMB, on the other hand, has been shown to be inherently resistant to inhibition via serum protease inhibitors which would explain the discrepancy between these two drugs. Regardless, we were pleased to see our GZMB pro-drug to have these effects on several lines in the presence of PSA as well as human prostate stromal cells which suggests that GZMB could have the capacity to disrupt the tissue architecture in a tumor which would yield a more comprehensive response. It is also worth noting that some cell lines (PC3 and LAPC4) were affected morphologically but not in terms of growth suggesting that these ECM interactions and their role in cell biology differs wildly between tumors.

To ensure whether GZMB's effects were solely due to ECM damage, we treated plates coated with ECM proteins from FBS with GZMB in a cell free context and then plated cells in serum free media once the GZMB was removed. We observed very similar effects on both cell lines which were sensitive to GZMB in co-culture experiments. Because GZMB was removed from the plate once the cells were added and that the cell morphology was very similar to that of the cells incubated on an empty plate with no ECM, it is reasonable to conclude that a majority the effects seen were due to ECM damage caused by GZMB proteolysis. It is possible; however, that the anti-proliferative effects seen on the cells were due to factors other than ECM damage, as it appears the cells grew slightly better than when incubated with GZMB directly.

Despite observing robust *in vitro* effects of these PSA-activated proteases, we observed no obvious antitumor effects of the drugs when injected IV or IT into LNCaP-bearing nude mice at any dose. While disappointing, this lack of efficacy may be due to not enough enzymatically active PSA being expressed by the tumors themselves as LNCaP xenografts, though PSA positive, do not produce near the levels of enzymatic PSA as human tumors. Because we used physiologically relevant concentrations of active PSA in our tissue culture experiments, we would expect these pro-drugs to be functional in the context of the prostate cancer microenvironment in a human. It is also possible that these drugs may not be able to generate anti-tumor effects by themselves and may need other means of chemotherapy to synergize with. While speculative, it is evident based on the observed *in vitro* effects that prostate cancer cells under duress will aggregate together in order to avoid undergoing anoikis or other negative effects. It is possible that combing our GZMB pro-drug with an agent that blocks these interactions may facilitate a synergistic anti-tumor effect. It is also possible that GZMB extracellular effects seems to be pro-inflammatory that a GZMB pro drug could be used to recruit local immune cells to the tumor and help facilitate an anti-tumor immune response. Our GZMB pro drug may be best used with immune check point inhibitors or other types of clinically approved cancer immunotherapy.

We successfully engineered two human enzymes to be activated by cancer-specific proteases not active in the bloodstream. These pro-drugs had favorable enzymatic activity characteristics while having some interesting anti-cancer activity *in vitro*. We are encouraged by this work as it elucidates a novel mechanism to regulate and engineer human proteases, one of the largest and most diverse classes of enzyme, to be activated by other proteases of interest. We postulate that the use of the EK inhibitory peptide and a known protease site could be used to modulate the activity of a number of proteases via proteolytic activation of a number of other target proteases. This may yield exciting developments in the fields of biotechnology, enzymology, and therapeutics. We are also encouraged by the idea of using therapeutic agents to generate a field effect that prevents tumor cells from thriving. Virtually all currently approved therapeutics directly, in one way or another, interact with the cancer cells. We have demonstrated a strategy to modify a microenvironment so that it changes tumor biology and generate an anti-tumor response. Should similar means be used successfully in *in vivo* models, these therapies may have the capacity to minimize recurrence of resistant cancer cells and combat the problem of tumor heterogeneity which has limited the efficacy of therapeutic strategies in extending and improving the quality of lives in patients.

## MATERIALS AND METHODS

### Design of PSA-activated GZMB and TRP proteins

In order to synthesize constructs for both PSA-activated proteins, careful consideration should be made as to the locus of the PSA substrate and the substrate itself. Because serine protease perturbation of ECM proteins requires higher concentrations of active enzyme, we chose the efficiently-cleaved, less specific PSA substrate KGISSQY. This peptide was inserted in between the inactivating EK peptide DDDDK and the N-terminal Isoleucine residue of both proteins. Thus, hydrolysis by PSA of either EK-PSA-GZMB or EK-PSA-TRP by PSA would yield exclusively the active GZMB and TRP enzymes. For EK-PSA-GZMB, a mutant containing a C-terminal reactive cysteine was evaluated. This cysteine moiety was introduced to allow for potential coupling of prostate specific targeting ligands to enhance toxicity of the protein. This modification had no effect on GZMB expression or catalytic activity (data not shown). Because EK, activates TRP, we chose to leave the native APF tripeptide as the N-terminus of EK-PSA-TRP. Figure 1 depicts this PSA-activation strategy.

### Cloning and mutagenesis of GZMB and TRP constructs

In order to express hGZMB in reasonable amounts using a mammalian expression system, the EK-C248 gene containing an N-Terminal enterokinase activation site was generated using by modification of the WT GZMB gene (Origene Plasmid SC321693). WT GZMB was amplified out of the respective vector, altered to contain the C-terminal cysteine, and flanked with EcoR1 restriction site and upstream vector sequence with the primer set F: CAGTGTGGTGGAATTCATGCAACCAATCCTGCTTC R: GATATCTGCAGAATTCTTAGTAGCGTTTCATGGTTTT. PCR was run using Accuprime Pfx Supermix (Thermo 12344-040) enzyme mix and run according to manufacturer's instructions with a Tm of 53°C. PCR product was purified on a 1.5% agarose gel after running at 100V for 45 minutes, excised, and isolated using a NucleoSpin Gel and PCR clean up kit (Machery-Nagel 740609). For transient expression in HEK-293T cells, WT GZMB-C248 (Eco R1) was inserted into the vector pcDNA 3.1 (+) (Thermo V790-20) using an InFusion HD Cloning kit (CloneTech 638909). The resulting gene-containing plasmid insert was then transformed into Stellar Competent *E. Coli* (CloneTech 636763) according to manufacturer's instructions. Colonies were picked, grown in 5mL LB containing 100 μg/mL Ampicillin for 8 hours, and expanded in 50mL LB containing 100 μg/mL. WT GZMB (pcDNA 3.1) was isolated using a PureLink® HiPure Plasmid Maxiprep Kit (K2100-06). DNA sequencing was performed using Sanger sequencing (T7 sequencing primer) via the Johns Hopkins Sequencing and Synthesis facility.

Because WT GZMB is toxic to HEK-293T cells, the protein must be expressed in an inactive conformation capable of being rapidly activated via proteolysis once expressed. For this reason, the native GE inhibitory di-peptide was replaced by an enterokinase cleavage site (as seen above). To mutate this cleavage site, GZMB-C248 (pcDNA3.1) was added to a Q5 Site Directed Mutagenesis (NEB E0554S) kit containing the primers F: CGACAAAATCATCGGGGGACATGAGG R: TCGTCGTCTGCATCTGCCCTGGGCAG. The reaction was run according to manufacturer's instructions with a Tm of 67°C and an extension time of 210 seconds. PCR product from this reaction was confirmed on a 1.5% agarose gel, ligated using the Q5 kit KLD enzyme mix, and transformed into NEB5α *E. Coli* according to manufacturer's instructions. EK-C248 (pcDNA3.1) was amplified, purified, and sequenced as described above. To convert this protein into a PSA-activated pro-drug, the PSA substrate KGISSQY was mutated in between the DDDDK EK peptide and the active sequence of GZMB. Using the mutagenesis primers F: CTCTCAGTACATCATCGGGGGACATGAG R: GAGATTCCTTTTTTGTCGTCGTCGTCTGC, the plasmid EK-PSA-GZMB (pcDNA 3.1) was generated using the aforementioned protocol with a Tm of 64°C. The plasmid was amplified, harvested, and sequenced as mentioned above. In order to make a mutant lacking the DDDDK piece but with a PSA substrate on the N-terminus (PSA-GZMB), we utilized the above mutagenesis protocol (EK-C248 pcDNA3.1 as a template) with the primer set F: AGGAATCTCCTCTCAGTACATCATCGGGGGACATGAGG R: TTATGATGATGATGATGATGTGCATCTGCCCTGGGCAG with a Tm of 67 degrees Celsius.

The plasmid containing the human PRSS1 gene containing the KGISSQY PSA substrate and a hexa-histidine purification tag was generated by first cloning the PRSS1 gene into pcDNA3.1. Using the InFusion protocol described above, we inserted flanking Eco RI restriction sites on the ends of the human PRSS1 gene (human cationic trypsin) (Human PRSS1 natural ORF mammalian expression plasmid, Sino Biological, HG10816-CH) using the primer set F:. CAGTGTGGTGGAATTCATGAATCCACTCCTGATCC R: GATATCTGCAGAATTCTTAGCTATTGGCAGCTATGG and a Tm of 52 degrees Celsius and an extension time of 210 seconds while following the recommended InFusion cloning protocol. Plasmid was transformed, grown, and harvested as described above. Next, we performed mutagenesis using the NEB method above in order to insert the KGISSQY sequence and a C-terminal Histidine tag. To insert the PSA sequence, the primer set F: CTCTCAGTACATCGTTGGGGGCTACAAC R: GAGATTCCTTTCTTGTCATCATCATCAAAGGG was used with a melting temperature of 61 degrees Celsius. For the mutagenesis respective to the Histidine tag, the set F: CACCACCATTAAGAATTCTGCAGATATCCAG R: GTGATGATGGCTATTGGCAGCTATGGTG and a Tm of 60 degrees Celsius. Lastly, to make a TRP mutant lacking the native EK piece but containing a PSA activations sequence (PSA-TRP) we mutagenized the EK-PSA-TRP pcDNA3.1 plasmid by deleting the DDDDK peptide. This was done using the primer set F: AAAGGAATCTCCTCTCAGTACATC R: AGCAAGAGCAGCTGCCAC with a Tm 64 degree Celsius. Like the GZMB constructs, all TRP mutants were sequenced using the Sanger method following isolation of the plasmid using a T7 promoter primer.

### Expression of recombinant proteins in HEK293T cells

HEK293T cells (ATCC® CRL-3216) were grown in DMEM w/ high glucose, 10% Fetal Bovine Serum, and supplemental L-glutamine. For all constructs, 5 × 10^6^ cells were plated in multiple T75 flasks and incubated for 48 hours at 37°C. To transiently transfect cultures, 25 μg of cDNA (per flask) was added to 1.2mL of OPTI-MEM low-serum media and mixed with 74 μL of FuGENE HD (Promega E2311). This mix was incubated for 10 minutes at room temperature, added to 20mL of DMEM media per T7, and incubated on cells overnight. To simplify the purification process, transfected cells were then washed once with 1X PBS (pH=7.4) and given serum-free, phenol-free DMEM w/ high glucose and L-glutamine. Cells were then incubated for at least 3 days. Supernatant was harvested before cell viability and attachment was compromised.

### Purification of recombinant GZMB and TRP proteins

Once supernatant from transiently transfected HEK cells was collected, cell debris was removed from via centrifugation at 8000 x g for 10 minutes. The clarified media was then concentrated 20-fold using Amicon Ultra Centrifugation Filters (UFC901008) and mixed 1:1 with 1X PBS (pH = 7.4). For GZMB-based pro-drugs (including PSA-GZMB), a cation exchange resin (Fractogel EMD SO_3_^-^ Millipore 116882) was used to isolate the protein. Because GZMB has an unusually high pI of 9.5, the protein will bind a negatively charged column at a higher affinity at pH 7.4. For TRP based constructs, a Histidine binding Nickel resin was used (His-Select Ni Affinity Resin, Sigma P6611). The immobilized proteins were then washed with either 5mL of PBS or PBS containing 20mM Imidazole respectively three times. GZMB and TRP pro-drugs were then eluted using PBS containing either 1M NaCl or 300mM Imidazole respectively. Protein elution was tracked during this process using UV spectroscopy at A280. Both constructs were then dialyzed to remove the components of the elution buffer and other small molecule contaminants. This was done by adding pooled fractions to a Slide-a-Lyzer Dialysis Cassette (MWCO 7K) (87722/87723) and dialyzing vs. PBS or PBS containing 1mM CaCl_2_ respectively. Dialyses were done at 500 fold excess volume and 1000 fold excess volume for 4 hours and 24 hours respectively. Final protein concentrations were determined using A280.

### PSA activation protease assays

To assess activation, purified PSA [ABD Serotec 7820-0504 (final concentration 10 μg/mL)] was added to a solution of EK-PSA-GZMB or EK-PSA-TRP in PBS at 37°C for the indicated time points. For Michaelis-Menten experiments, pre-incubations of EK-PSA-GZMB and EK-PSA-TRP with PSA were done overnight and 4 hours respectively.

To determine the enzymatic activity of GZMB, a fluorescent based GZMB assay kit (Biovision K168-100) with the substrate IETD-AFC, was used according to the manufacturer's instructions. Following a one hour incubation with GZMB at 37°C, fluorescence was measured at 380 nm excitation and 500 nm emission wavelengths. Concentrations of AFC released were calculated based off a standard curve of diluted AFC. For Michaelis-Menten kinetics of GZMB, a chromogenic substrate was used to measure catalytic activity of the protein. Specifically, Ac-IETD-pNA (AG Scientific G-1085) was diluted in DMSO to a stock concentration of 25 mM and diluted to various concentrations. EK-PSA-GZMB solution was then added 1:1 and read at 405 nm at various time points. For EK-PSA-TRP activity and kinetic experiments, z-GPR-pNA (Sigma C2276) was diluted in DMF to a concentration of 2 mM (for activity experiments) and mixed 1:1 with protein solutions. Solutions were read at 405 nm after 15 min for activity assays and at various time points to determine kinetic parameters. For all assays, the extinction coefficient 13500 M^-1^cm^-1^ L was used to determine the concentration of released pNA. All readings were corrected to background florescence/absorbance. Activity readings are represented as amount of substrate process per minute per mg of enzyme added.

### SDS PAGE and western blot analysis

In order to confirm recombinant proteins were correctly sized, SDS PAGE was performed using a BioRad gel electrophoresis system. Samples containing recombinant EK-PSA-GZMB/TRP proteins were mixed 1:1 with 4X reducing SDS PAGE Sample Buffer and boiled for 5 minutes. Samples were then loaded and run at 150V until fully migrated. For protein staining, the gel was then washed 2-3 times with RO H_2_O and stained for one hour with SimplyBlue Safe Stain solution (Thermo LC6060). To de-stain samples were washed in RO H_2_O overnight. For Western blotting, samples were run on a non-reducing gel and were transferred to PVDF Immuno-Blot membrane (BioRad 1620177) for 1 hour at 100V and incubated for one hour in TBST containing 5% milk. To monitor removal of the DDDK peptide, an anti-DDDDK polyclonal antibody (Abcam 1162) was diluted to 1:5000 in TBST/milk and incubated with the membrane overnight at 4°C. The next day, the membrane was washed 5X with TBST and incubated with an anti-rabbit HRP-linked IgG (Cell Signaling #70762) diluted at 1:10000 in TBST/milk for one hour at 4°C. The blot was then washed 5X with TBST and incubated with chemiluminescent substrate solution (Thermo 34077) diluted according to manufacturer's instructions. The blots were then developed at specified time points.

### Edman degradation of EK-PSA-GZMB

In order to demonstrate site-specific cleavage and activation of EK-PSA-GZMB by PSA, EK-PSA-GZMB was treated with plus PSA or buffer as described above overnight at 37°C. Both samples were then run on an SDS PAGE gel and transferred to a PVDF membrane as described above. The blot was then dried at room temperature for an hour and stained with SimplyBlue stain to visualize proteins. Sample was then de-stained using a solution of methanol (40% v/v) and acetic acid (10% v/v). Bands corresponding to the molecular weight of EK-PSA-GZMB were cut and stored in 1.7mL microfuge tubes. Edman degradation was performed by the Johns Hopkins Sequencing and Synthesis facility. Analysis was done on the three most N-terminal amino acids for each sample.

### Cell culture, viability, and microscopy experiments

Prostate cancer cell lines were from ATCC and were grown in RPMI 1640 medium containing 10%FBS and supplemental L-glutamine. Normal prostate stromal cells were provided by Dr. N. Brennen, Johns Hopkins and maintained under similar conditions. For serum free conditions, to maintain cell viability and growth, LNCaP, and CWR22 Rv1 cells were plated on a 96 well plate pre-coated with FBS (100μL RPMI with 10% serum per well for 4 hours at 37°C) in phenol-free, serum-free RPMI 1640 with L-glutamine and B27 serum supplement (Thermo 17504). Cells were plated at 5000 cells per well unless stated otherwise. After 24 hours cells were treated with various doses of EK-PSA-GZMB and EK-PSA-TRP plus or minus PSA at a final concentration 5 μg/mL unless otherwise indicated. Control cells were treated with the respective dialysis buffer only. For GZMB-attachment experiments, medium with or without 10% FBS plus or minus 1μM GZMB was placed in 96 well plates. Solutions were then removed and cells were plated in serum-free RPMI containing B27. To The MTT assay (Promega G4000) was used according to manufacturer's instructions to determine cell viability. Cell number was calculated based off a standard absorbance curve as previously described [9]. To demonstrate altered cell morphology, cells were photographed using a Nikon TE200 fluorescence microscope with the Metamorph software package at indicated magnifications.
